# Clinical features of lupus enteritis: a single-center retrospective study

**DOI:** 10.1186/s13023-021-02044-4

**Published:** 2021-09-26

**Authors:** Long Chen, Qin He, Man Luo, Yuxiao Gou, Dan Jiang, Xiaoqin Zheng, Gaowu Yan, Fang He

**Affiliations:** 1 Department of Rheumatology and Immunology, Suining Central Hospital, No.127, West Desheng Rd., Chuanshan District, Suining, Sichuan Province China; 2 Department of Radiology, Suining Central Hospital, No.127, West Desheng Rd., Chuanshan District, Suining, Sichuan Province China; 3 Department of Scientific Research Management, Suining Central Hospital, No.127, West Desheng Rd., Chuanshan District, Suining, Sichuan Province China

**Keywords:** Systemic lupus erythematosus, Lupus enteritis, Mesenteric vasculitis, Target sign, Comb sign, Abdominal pain, High-dose glucocorticoids, Immunosuppressant, Abdominal computed tomography

## Abstract

**Background:**

Lupus enteritis (LEn) is a rare complication of systemic lupus erythematosus (SLE). Timely diagnosis and treatment of LEn are necessary to prevent the most serious consequences — intestinal perforation, gastrointestinal bleeding, and death. We compared the clinical features of SLE patients with and without LEn.

**Methods:**

The clinical data of LEn inpatients at Suining Central Hospital from July 2012 to June 2020 were examined. These LEn patients were matched (1:2 ratio) with concurrently hospitalized SLE patients who did not have LEn. The two groups were compared using multivariate logistic regression.

**Results:**

We compared SLE inpatients with LEn (n = 43) and SLE inpatients without LEn (n = 86) at our institution. Multivariate logistic regression showed that ascites (odds ratio [OR]: 9.961, 95%CI: 2.215–44.802, *P* = 0.003), hydronephrosis (OR: 28.060, 95%CI: 2.303–341.962, *P* = 0.009), leukopenia (OR: 5.890, 95%CI: 1.813–19.135, *P* = 0.003), reduced complement C3 level (OR: 4.791, 95%CI: 1.605–14.300, *P* = 0.005), and elevated immunoglobin (Ig)A level (OR: 4.040, 95%CI: 1.307–12.487, *P* = 0.015) were independently associated with LEn. Within the LEn group, abdominal pain was the most common abdominal symptom (88.4%), and increased mesenteric fat attenuation (74.4%) and bowel wall thickening (58.1%) were the most common computed tomography (CT) findings. Most LEn patients (88.4%) required high-dose glucocorticoid therapy (≥ 80 mg methylprednisolone/day), and cyclophosphamide was the most commonly used immunosuppressant (62.8%).

**Conclusions:**

Abdominal pain was the most common clinical symptom of LEn. Abdominal CT provides important information for detection and diagnosis of LEn. Ascites, hydronephrosis, leukopenia, hypocomplementemia (C3), and increased IgA were independently associated with LEn.

**Supplementary Information:**

The online version contains supplementary material available at 10.1186/s13023-021-02044-4.

## Introduction

Systemic lupus erythematosus (SLE) is an autoimmune disease that can damage multiple organs and organ systems. SLE-mediated damage to the digestive system can manifest as oral ulcers, pseudo intestinal obstruction, protein-losing enteropathy, liver damage, autoimmune pancreatitis, lupus enteritis (LEn), and other complications [1, 2]. LEn is a rare digestive symptom complication of SLE that is also referred to as lupus mesenteric vasculitis, gastrointestinal vasculitis, or acute gastrointestinal syndrome [3]. Pain is the main abdominal symptom of LEn, and it may be accompanied by diarrhea and vomiting [4]. LEn should be suspected in SLE patients who have intestinal symptoms after exclusion of infection.

Abdominal computed tomography (CT) is the main method used to diagnose LEn. The typical CT manifestations are bowel wall thickening (“target sign”) and mesenteric vasodilation (“comb sign”) [3, 4]. Digestive endoscopy has low sensitivity in the detection of LEn, and more than half of these patients have normal endoscopy findings [4]. Pathological findings include cellular infiltration of the submucosal and muscular layers, with or without edema or vasculitis [4].

We retrospectively analyzed the clinical characteristics of 43 SLE patients with LEn and 86 SLE patients without LEn who were admitted to the inpatient division of Suining Central Hospital (Sichuan, China) to identify factors associated with LEn.

## Methods

### Screening of the LEn and non-LEn groups

**Fig. 1:**
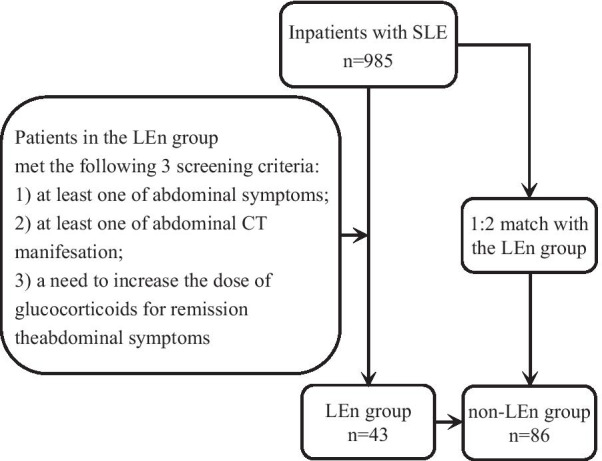
The process of screening patients in the LEn and non-LEn groups

Inpatients with LEn (LEn group) who were admitted to the Suining Central Hospital (Sichuan Province, China) from July 2012 to June 2020 were included. All patients met the criteria for LEn provided in the 1997 classification of SLE by the American College of Rheumatology (ACR) [5] and also had the following three conditions: (i) abdominal symptoms and at least one of the following: abdominal pain, diarrhea, bloating, nausea, or vomiting; (ii) at least one of the following three imaging manifestations: bowel wall thickening, mesenteric vasodilation, or increased attenuation of mesenteric fat [3, 4, 6]; and (iii) no relief from digestive symptoms following use of acid inhibitors, mucosal protective agents, gastrointestinal motility drugs, or antibiotics, but successful alleviation after use of an increased dosage of glucocorticoids. Then, two non-LEn patients were selected to match with each LEn patient in the consecutive series. All patients in the non-LEn group were also inpatients who met the 1997 classification of SLE from the ACR (Fig. 1).

### Data collection

The following parameters were recorded for each patient in both groups: age; sex; disease course; age at disease onset; presence of pleural effusion, pericardial effusion, ascites, hydronephrosis, fever, arthritis/arthralgia, skin rash, lupus nephritis, leukopenia, anemia, and thrombocytopenia; hypocomplementemia (C3, C4); levels of immunoglobulin (Ig) A, IgG, and IgM; and results of an autoantibody panel. Pleural effusion, pericardial effusion, and ascites were confirmed by ultrasonography or CT. An axillary temperature above 37.5 °C was considered to be elevated. Skin rashes were classified as butterfly rash, discoid rash, vasculitis-like rash, erythema hyperemia, or frostbite-like rash. Lupus nephritis was defined by a 24-h urinary protein level (total urine protein) above 0.5 g or positive biopsy results. A white blood cell count below 3.5 × 10^9^/L was considered leukopenia; a hemoglobin level below 110 g/L (females) or below 120 g/L (males) was considered anemia; a total platelet count below 100 × 10^9^/L was considered thrombocytopenia; a plasma albumin level below 40.0 g/L was considered hypoalbuminemia; a C3 level below 0.7 g/L and a C4 level below 0.1 g/L were considered low; and increased Ig levels were above 4.2 g/L for IgA, 17.4 g/L for IgG, and 2.8 g/L for IgM.

An indirect immunofluorescence assay was used to detect anti-ds-DNA antibody and immunoblotting was used to detect anti-nucleosome antibody, anti-ribosomal P protein antibody, anti-histone antibody, anti-Smith antibody, anti-SSA antibody, anti-SSB antibody, and anti-proliferating cell nuclear antigen antibody. For all the patients in the LEn group, abdominal symptoms (pain, diarrhea, bloating, nausea, and vomiting), abdominal CT findings (bowel wall thickening, comb sign, increased attenuation of mesenteric fat, bowel distension, and ascites), and drug regimens (maximum dosage of glucocorticoids and use of immunosuppressants, intravenous gamma globulin, and antibiotics) were recorded.

### Follow-up of the LEn group

Only patients in the LEn group were followed up, and the two endpoints were patient survival as of the last follow-up and relapse of LEn. Follow-up consisted of inquiries using the hospital information system and direct telephone contact with the patient or relatives to determine the occurrence of wither endpoint. The cut-off date for the follow-up was 31 January 2021.

### Statistical analysis

Continuous variables are reported as mean ± standard deviation (SD) or median (interquartile range, IQR) and were compared by using a Student's *t*-test or the Mann–Whitney *U* test, as appropriate. Discrete variables are presented as the frequency (percentage) and were compared using the chi-square test or Fisher’s exact test. Variables in which the univariate analysis indicated the significance of the difference was below 0.2 were included in multivariate logistic regression analysis (forward selection method) to screen for factors independently associated with LEn. All data analyses were performed using SPSS version 22.0 (IBM, Armonk, NY). For all statistical analyses, differences were deemed statistically significant when the two-sided *P* value was below 0.05.

## Results

### Screening of the LEn and non-LEn groups

A total of 985 individuals with SLE were inpatients at our hospital from July 2012 to June 2020. After screening for eligibility, we examined 43 patients in the LEn group and 86 matched patients in the non-LEn group. The overall incidence of LEn among SLE patients at our institution was approximately 4.4% (Fig. 1).

### Comparisons of clinical data in the LEn and non-LEn groups

The comparison of general information (the sex ratio, age at screening, age of onset and course of the disease) collected from these two groups of patients indicated no significant difference. This indicated these two groups were comparable, and allowed us to perform meaningful comparisons of other clinical data (Table 1).

**Table 1:** General information of the LE and non-LEn groups

	LE group	Non-LEn group	*P*-value
Male sex n (%)	5 (11.63)	3 (3.49)	0.116^c^
Age at screening (years)^a^	40.0 (19.0)	39.5 (23.2)	0.719^d^
Disease duration (years)^a^	3.0 (7.7)	3.7 (7.1)	0.589^d^
Age of disease onset(years)^b^	33.2 ± 11.5	32.8 ± 13.6	0.201^e^

^a^Median (interquartile range, IQR)

^b^Mean ± standard deviation (SD)

^c^Fisher’s exact test;

^d^Mann-Whitney *U* test

^e^Student's t test

Comparisons of the clinical data in the two groups showed that pleural effusion, ascites, hydronephrosis, skin rash, lupus nephritis, leukopenia, hypoalbuminemia, hypocomplementemia (C3 and C4), increased IgA level, and positivity for anti-nucleosome-ANA antibodies were significantly more common in the LEn group (all *P* < 0.05; Table 2).

**Table 2:** Baseline characteristics of the LE and non-LEn groups

	LE group n (%)	Non-LEn group n (%)	χ^2^	*P* value
*Clinical manifestations*				
Pericardial effusion	7 (16.3)	14 (16.3)	0	1.000^a^
Pleural effusion	15 (34.9)	14 (16.3)	5.694	0.017^a^
Ascites	12 (27.9)	3 (3.5)	16.634	< 0.001^a^
Hydronephrosis	6 (14.0)	1 (1.2)	–	0.003^b^
Fever	10 (23.3)	16 (18.6)	0.385	0.535^a^
Arthritis/arthralgia	14 (32.6)	20 (23.3)	1.278	0.258^a^
Rash	18 (41.9)	21 (24.4)	4.135	0.042^a^
Lupus nephrits	19 (44.2)	22 (25.6)	4.576	0.032^a^
*Biological features*				
Leukopenia	10 (23.3)	8 (9.3)	4.649	0.031^a^
Anaemia	24 (55.8)	38 (44.2)	1.553	0.213^a^
Thrombocytopenia	12 (27.9)	17 (19.8)	1.090	0.297^a^
Hypoalbuminemia	30 (69.8)	35 (40.7)	9.691	0.002^a^
*Immunological features*				
Hypocomplementemia (C3)	37 (86.0)	44 (51.2)	14.931	< 0.001^a^
Hypocomplementemia (C4)	23 (53.5)	26 (30.2)	6.582	0.010^a^
Elevated IgA	13 (30.2)	12 (14.0)	4.862	0.027^a^
Elevated IgG	12 (27.9)	28 (32.6)	0.290	0.590^a^
Elevated IgM	0 (0.0)	4 (4.7)	–	0.302^b^
Anti-ds-DNA	19 (44.2)	26 (30.2)	2.457	0.117^a^
Anti-nu antibody positivity	18 (41.9)	19 (22.1)	5.476	0.019^a^
Anti-PP antibody positivity	20 (46.5)	25 (29.1)	3.839	0.050^a^
Anti-P antibody positivity	14 (32.6)	15 (17.4)	3.759	0.053^a^
Anti-Sm antibody positivity	13 (30.2)	17 (19.8)	1.759	0.185^a^
Anti-PCNA antibody positivity	1 (2.3)	1 (1.2)	–	1.000^b^
Anti-SSA antibody positivity	34 (79.1)	57 (66.3)	2.257	0.133^a^
Anti-SSB antibody positivity	8 (18.6)	17 (19.8)	0.025	0.875^a^

Anti-ds-DNA, anti-double-stranded DNA; Anti-nu, anti-nucleosome; Anti-P, anti-histone antibody; Anti-PCNA, anti-proliferating cell nuclear antigen; Anti-PP, anti-ribosomal P protein; Ig: immunoglobulin

^a^Chi-square test

^b^Fisher’s exact test

### Analysis of factors associated with LEn

We used multivariable analysis with forward selection to further analyze variables that had *P* values were below 0.2 in the analysis above. The results indicated that ascites, hydronephrosis, leukopenia, decreased C3 level, and increased IgA level were independently associated with LEn (Table 3).

**Table 3:** Screening independent associated factors of LEn by multivariate logistic regression analysis (forward method)

	B	SE	*P*-value	OR (95%CI)
Ascites	2.299	0.767	0.003	9.961 (2.215–44.802)
Hydronephrosis	3.334	1.276	0.009	28.060 (2.303–341.962)
Leukopenia	1.773	0.601	0.003	5.890 (1.813–19.135)
Hypocomplementemia (C3)	1.567	0.558	0.005	4.791 (1.605–14.300)
Increased IgA	1.396	0.576	0.015	4.040 (1.307–12.487)

95% CI, 95% confidence interval; B, coefficient value; IgA, immunoglobulin A; OR, odds ratio; SE, standard error

### Gastrointestinal symptoms and imaging manifestations in patients with LEn

**Fig. 2:**
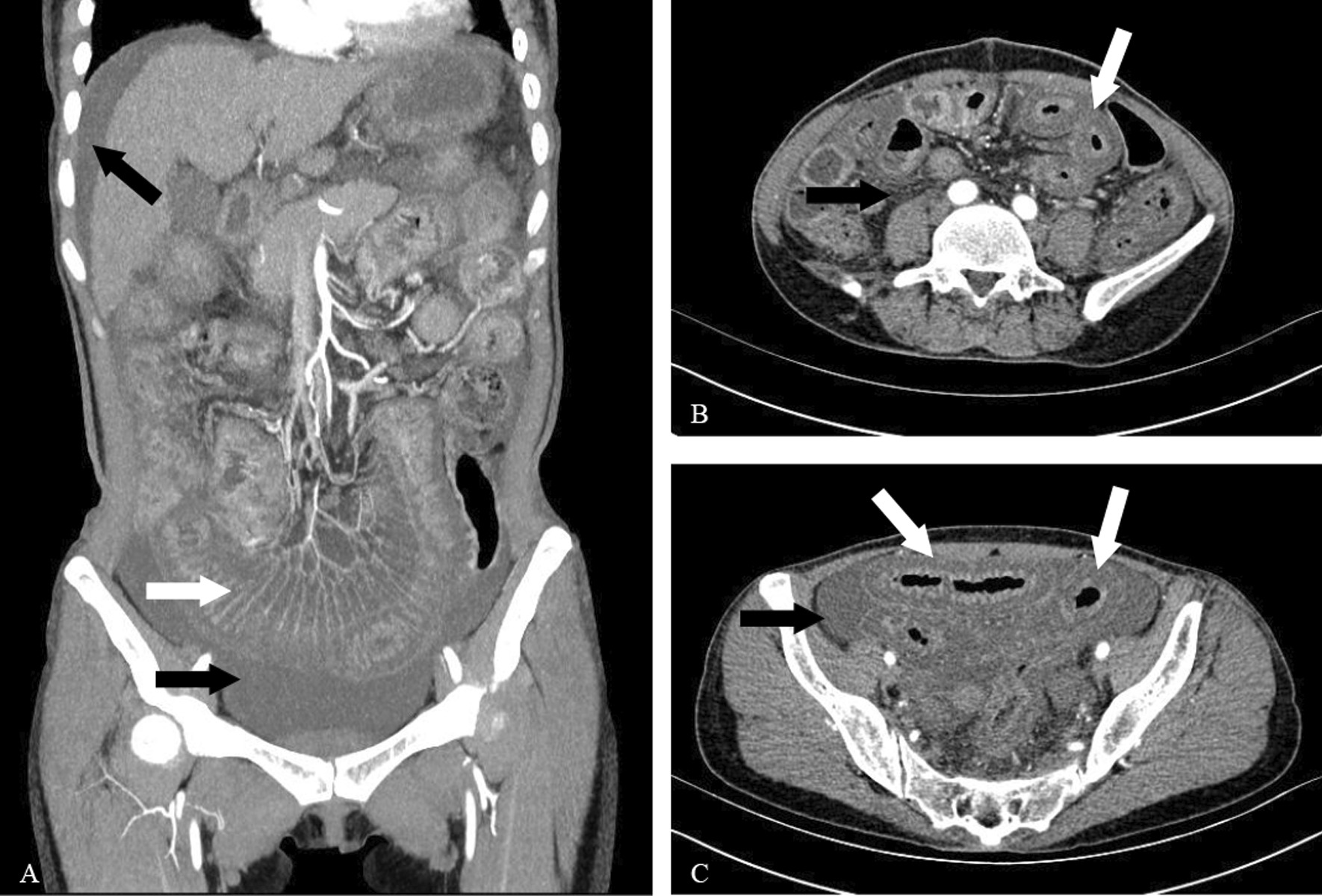
The CT manifestation of lupus enteritis. **A**: massive fluid in the abdominal cavity (the black arrow) and engorgement of mesenteric vessels (comb sign, the white arrow); **B**: increased attenuation of mesenteric fat (the black arrow) and the bowel wall thickening (target sign, the white arrow); **C**: massive fluid in the abdominal cavity (the black arrow), the bowel wall thickening and bowel dilatation (the white arrow)

The common abdominal symptoms in patients with LEn were abdominal pain (38/43, 88.4%), nausea (23/43, 53.5%), vomiting (21/43, 48.8%), abdominal distension (21/43, 48.8%), and diarrhea (17/43, 39.5%). Abdominal CT findings showed that more than half of patients with LEn had increased attenuation of mesenteric fat (32/43, 74.4%) and bowel wall thickening (25/43, 58.1%), although the mesenteric vasodilation (11/43, 25.6%) and ascites (12/43, 27.9%) were less common (Table 4, Fig. 2).

**Table 4:** Clinical manifestation and treatment in the LEn group

Clinical manifestation/treatment	n (%)
*Gastrointestinal symptoms*	
Abdominal pain	38 (88.4)
Diarrhea	21 (48.8)
Abdominal distension	21 (48.8)
Nausea	23 (53.5)
Vomiting	17 (39.5)
*CT manifestation*	
Bowel wall thickening	25 (58.1)
Engorgement of mesenteric vessels	11 (25.6)
Increased attenuation of mesenteric fat	32 (74.4)
Bowel dilatation	17 (39.5)
Ascites	12 (27.9)
*Therapeutic intervention*	
Glucocorticoid (MP ≥ 80 mg/day)	38 (88.4)
Immunosuppressant	
CTX	27 (62.8)
MMF	9 (20.9)
IVIG	7 (16.3)
Antibiotics	23 (53.5)

CTX, cyclophosphamide; IVIG, intravenous immunoglobulin; MMF, mycophenolate mofetil; MP, methylprednisolone

### Treatment and follow-up

All LEn patients received intravenous methylprednisolone to induce remission, with a minimum dosage of 40 mg/day. Thirty-six patients (36/43, 83.7%) received combined immunosuppressive therapy (intravenous of cyclophosphamide at 0.5–1.0 mg/m^2^/month or oral mycophenolate mofetil at 1.5–2.0 g/day) after LEn remission. Among these 36 patients, the major systemic lesions were lupus nephritis (19/43, 44.2%), neuropsychiatric lupus (5/43, 11.6%), autoimmune hemolytic anemia (3/43, 7.0%), diffuse alveolar hemorrhage (1/43, 2.3%), and lupus hepatitis (1/43, 2.3%). Approximately half of the patients with LEn (23/43, 53.5%) also received antibiotics during treatment, and 7 patients (7/43, 16.3%) received intravenous Ig (IVIG) (Table 4).

The median follow-up time in the LEn group was 58 months (range: 14–92 months). During follow-up, one LEn patient died of *Pneumocystis carinii* pneumonia (patient no. 16, Additional file 1: Table 1; Additional file 2: Figure 1) and one patient had LEn recurrence (identified as patient no. 23 and also as patient no. 40; Additional file 1: Table 1).

## Discussion

SLE is a relatively common autoimmune disease that can affect many organs. Organ damage mediated by autoantibodies and autoreactive T lymphocytes are the main features of SLE. The clinical manifestations are highly heterogeneous, and SLE affects many organ systems throughout the body [7]. Damage to the digestive system is very common in these patients because the SLE itself can lead to recurrent oral mucosal ulcers, lupus hepatitis, autoimmune pancreatitis, protein-losing enteropathy, and LEn. Moreover, gastrointestinal reactions and liver dysfunction can be caused by the use of non-steroidal anti-inflammatory agents, glucocorticoids, immunosuppressants, and other drugs [1, 2, 8–12]. LEn is a rare disease that is secondary to SLE whose incidence varies according to geography and race. A literature review by Ju et al. [3] reported that the global incidence of LEn in SLE patients was approximately 0.2 to 9.7%.

The risk factors for the LEn among SLE patients have not been fully delineated. Lee et al. [13] analyzed 175 SLE patients and assigned them to three groups: (i) SLE + LEn with abdominal pain, (ii) SLE alone with abdominal pain, and (iii) SLE alone without abdominal pain. They showed that leukopenia was more common in the first group than in the other two groups, in agreement with our findings. A reduced level of complement C3 is often a sensitive indicator of active SLE. We found that a reduced C3 level was more common in SLE patients with LEn than in those without LEn. However, previous studies [13, 14] reported inconsistent findings regarding the association between LEn and active SLE. Buck et al. [14] reported that lupus mesenteric vasculitis occurred only in patients with active disease (SLE disease activity index [SLEDAI] score > 8), but Lee et al. [13] showed that the SLEDAI score did not differ significantly between SLE patients with abdominal pain and LEn and SLE patients with abdominal pain but no LEn. Their findings [13] suggested that the SLEDAI score may be unsuitable for disease assessment and treatment decisions in patients with LEn. Some other studies reported the co-occurrence of lupus-related urinary system damage and LEn [15–18], and the manifestations of urinary tract damage in these patients included hydronephrosis and lupus cystitis. Approximately 14.0% (6/43) of the patients in our LEn group had hydronephrosis, significantly more than in the non-LEn group. Our multivariate regression analysis confirmed that hydronephrosis was significantly and independently associated with LEn, consistent with the conclusions in several previous studies [15–18].

Interestingly, few previous studies have examined the serum levels of IgA, IgG, and IgM in patients with LEn. We found that the level of serum IgA was significantly greater in the LEn group than in the non-LEn group, and our multivariate regression analysis showed that an increased level of serum IgA was associated with LEn. IgA functions in the mucosal immune barrier and in resistance to pathogenic microorganisms. The intestine is the largest human organ with mucosal tissues, and is the main organ that produces and secretes IgA. IgA also plays an important role in maintaining the stability of intestinal microecology [19, 20]. Hence, the connection between increased IgA level and LEn seems biologically plausible, but further studies are necessary to confirm the clinical significance and underlying mechanisms.

Similar to the findings of previous studies [11–16], the clinical symptoms in our LEn patients were not specific, and abdominal pain was the most common symptom. Approximately 90% of our LEn patients had abdominal pain of varying severity, and some patients also had nausea, abdominal distension, diarrhea, and vomiting. Clinicians may suspect LEn when abdominal symptoms occur in SLE patients, and this highlights the need for timely abdominal CT, especially enhanced abdominal CT which is more sensitive in the detection of intestinal abnormalities. There are three typical abdominal CT findings in patients with LEn, and these can appear alone or concurrently: (*i*) bowel wall thickening (> 3.0 mm), which leads to separation of the mucosa and muscle layers and appearance of the “target sign”; (*ii*) mesenteric vasodilation with appearance of the “comb sign”; and (*iii*) increased attenuation of mesenteric fat [16, 21–25]. The co-occurrence of the “target sign” and “comb sign” is particularly specific to LEn, and can be used to establish a diagnosis. In addition to CT manifestations, ultrasonography and magnetic resonance enterography (MRE) can also be used to diagnose LEn. For example, Demiselle et al. [26] described a patient with LEn who had characteristic intestinal wall edema and ascites based on ultrasonography. Cicero et al. [27] used MRE to observe a formation with the appearance of a thumb print caused by bowel ischemia and bowel wall edema in a patient with LEn.

Due to the lack of prospective randomized controlled clinical trials, there are currently no available guidelines or recommendations for the treatment of LEn. However, previous studies [3, 4] reported that most LEn patients achieved remission following high-dose glucocorticoids, with or without the addition of immunosuppressive therapy. The major immunosuppressants used in these patients are cyclophosphamide, azathioprine, and mycophenolate mofetil [4]. Early case reports found that most patients with LEn received glucocorticoids with cyclophosphamide [28–30]. Lian et al. [31] retrospectively analyzed patients with SLE and acute gastrointestinal syndrome and showed that the combined use of cyclophosphamide and glucocorticoids significantly improved patient prognosis. In our study, all patients with LEn were relieved of abdominal symptoms by high-dose glucocorticoids. However, there are no guidelines regarding the use of immunosuppressive agents combined with glucocorticoids for these patients. In clinical practice, a comprehensive assessment of the patient's complications, such lupus nephritis, neuropsychiatric lupus, and other important organ damage, is necessary to guide treatment decisions, such as combined immunosuppressive therapy. About half of our LEn patients also had active lupus nephritis, and some other patients had neuropsychiatric lupus, autoimmune hemolytic anemia, diffuse alveolar hemorrhage, and other complications. Hence, more than half of our patients with LEn received glucocorticoids in combination with cyclophosphamide. Approximately 20.9% of these patients received mycophenolate with glucocorticoids, and 16.3% received glucocorticoids alone.

Death from LEn is rare, and only one patient in our LEn group died during the follow-up. This patient had recurrent abdominal pain for more than two months that was rapidly relieved by treatment with high-dose glucocorticoids with cyclophosphamide, but she unfortunately died of *Pneumocystis carinii* pneumonia. In addition, only one of our patients experienced LEn recurrence. This patient initially received glucocorticoids alone to induce remission, but received combined treatment with glucocorticoids and cyclophosphamide after recurrence. Maruyama et al. [32] showed that LEn was likely to recur, and reported that 29% (5/17) of their LEn patients experienced recurrence. These authors suggested that bowel wall thickness exceeding 9.0 mm may be a predictor of recurrence. They also reported [32] that among the 5 patients with recurrence, 2 patients initially received glucocorticoids alone.

Our study was limited by the small sample size and the single-center and retrospective design. Because it was a retrospective study, there were some missing data that made it impossible to analyze certain data, such as antiphospholipid antibodies and intestinal ultrasound results. Second, due to our lack of baseline data (before onset of LEn), we could not make predictions about related risk factors. Therefore, further studies of the clinical characteristics of LEn should use a larger more rigorous prospective cohort design, or even a multi-center randomized controlled clinical trial.

## Conclusions

LEn is a rare complication of SLE, and abdominal pain is the most common clinical symptom. Abdominal CT should be performed in SLE patients who report abdominal pain to confirm the presence of LEn, especially in patients who have other factors independently associated with LEn. Timely administration of high-dose glucocorticoid therapy is effective and can improve the prognosis of these patients. The decision of whether to combine a glucocorticoid with an immunosuppressive agent requires comprehensive consideration of the comorbidities of individual LEn patients. More high-quality registration studies are needed to focus on this rare complication of SLE.

## Supplementary Information

**Additional file 1: Table 1.** Abdominal symptoms, CT manifestations, and treatments of patients with lupus enteritis.

**Additional file 2: Figure 1.** Lung CT results in the patient with lupus enteritis who died from *Pneumocystis carinii* pneumonia.

## Data Availability

The datasets used and/or analyzed during the current study are available from the corresponding author on reasonable request.
