# Supplementary material for: Clinical features of lupus enteritis: a single-center retrospective study
Source: Orphanet J Rare Dis. 2021 Sep 26;16:396. doi: 10.1186/s13023-021-02044-4 (PMC8474739; doi:10.1186/s13023-021-02044-4)
Supplement: Supplementary file 2 — Additional file 2: Figure 1. Lung CT results in the patient with lupus enteritis who died from Pneumocystis carinii pneumonia. [file 13023_2021_2044_MOESM2_ESM.pptx]

## Slide 1
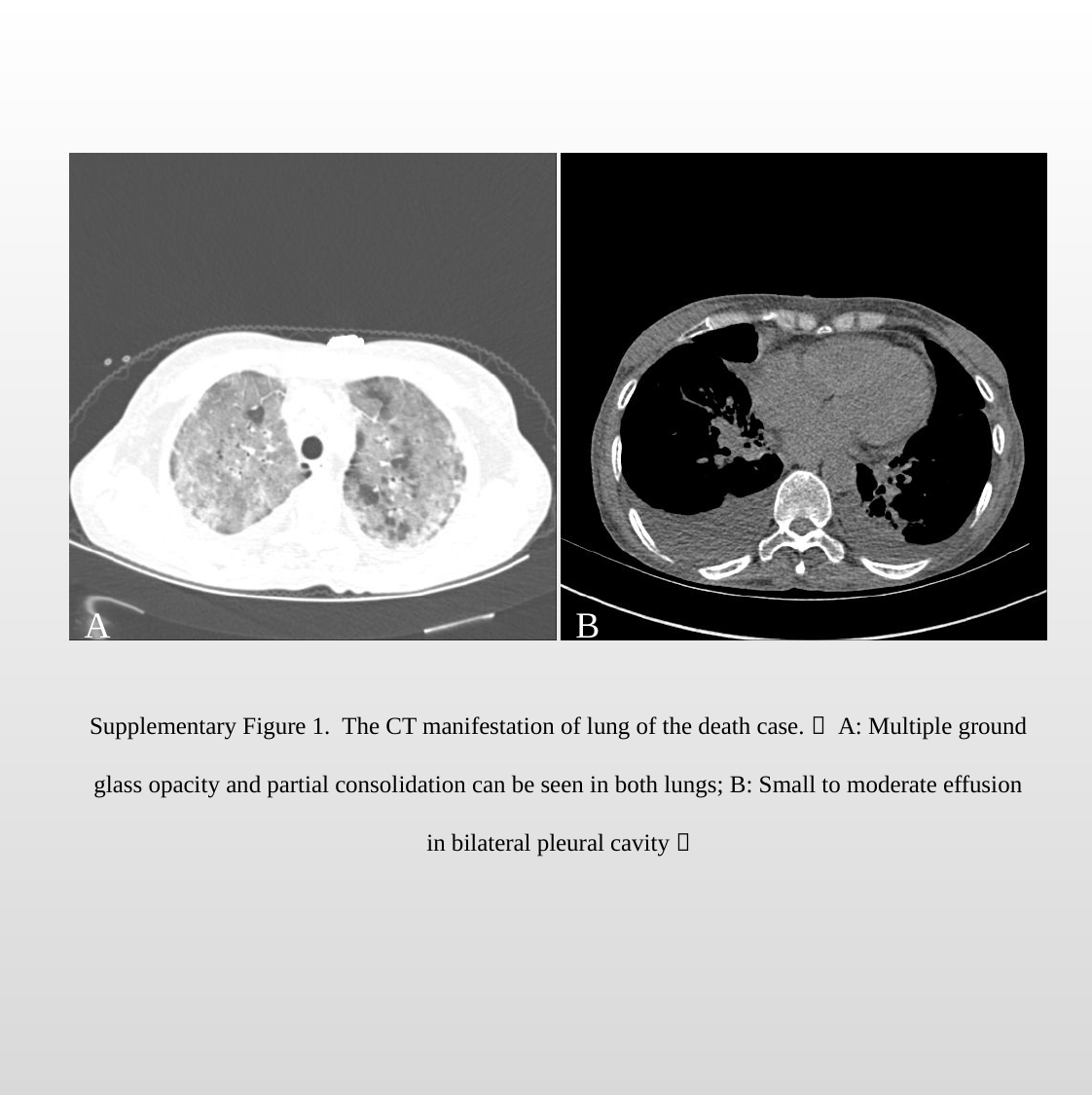

A
B
Supplementary Figure 1. The CT manifestation of lung of the death case.（ A: Multiple ground glass opacity and partial consolidation can be seen in both lungs; B: Small to moderate effusion in bilateral pleural cavity）
